# A comparison of two collapsing methods in different approaches

**DOI:** 10.1186/1753-6561-8-S1-S8

**Published:** 2014-06-17

**Authors:** Carmen Dering, Arne Schillert, Inke R König, Andreas Ziegler

**Affiliations:** 1 Institut für Medizinische Biometrie und Statistik, Universität zu Lübeck, Universitätsklinikum Schleswig-Holstein, Campus Lübeck, Ratzeburger Allee 160, Haus. 24, 23562 Lübeck, Germany; 2 Zentrum für Klinische Studien, Universität zu Lübeck, Ratzeburger Allee 160, Haus. 2, 23562, Lübeck, Germany

## Abstract

Sequencing technologies have enabled the investigation of whole genomes of many individuals in parallel. Studies have shown that the joint consideration of multiple rare variants may explain a relevant proportion of the genetic basis for disease so that grouping of rare variants, termed *collapsing*, can enrich the association signal.

Following this assumption, we investigate the type I error and the power of two proposed collapsing methods (combined multivariate and collapsing method and the functional principal component analysis [FPCA]-based statistic) using the case-control data provided for the Genetic Analysis Workshop 18 with knowledge of the true model. Variants with a minor allele frequency (MAF) of 0.05 or less were collapsed per gene for combined multivariate and collapsing. Neither of the methods detected any of the truly associated genes reliably. Although combined multivariate and collapsing identified one gene with a power of 0.66, it had an unacceptably high false-positive rate of 75%. In contrast, FPCA covered the type I error level well but at the cost of low power. A strict filtering of variants by small MAF might lead to a better performance of the collapsing methods. Furthermore, the inclusion of information on functionality of the variants could be helpful.

## Background

In recent years, several technologies have been released that allow the sequencing of whole genomes of large groups of individuals. Millions of rare mutations in the genome can be identified, and both common and rare variants can be analyzed jointly. This technology also enables analyses following the common disease-rare variant (CD-RV) hypothesis, which states that disease etiology is caused by multiple rare variants with moderate to high penetrances [1]. Studies have shown that the joint consideration of multiple rare variants may partly explain the genetic basis of disease [2]. To this end, grouping of rare variants in a region of interest (ROI), such as a gene, could enrich the association signal. Several approaches, termed *collapsing methods *or *burden methods*, incorporate this concept (for reviews, see [3-5]).

In this study, we compare two collapsing methods that use the genetic information in different ways. Specifically, we consider the combined multivariate and collapsing (CMC) method [6] and functional principal component analysis (FPCA)-based statistic [7] to test for groupwise association with the simulated disease status in unrelated individuals. For comparison, we used the case-control data provided for the Genetic Analysis Workshop 18 (GAW18) with knowledge of the answers.

## Methods

### Functional principal component analysis-based statistic

Luo *et al *[7] use the genome continuum model [8] and principal component analysis (PCA) as the basis for their test statistic. After scaling each ROI to the interval of [0, 1] a ROI-wise integral function *f *of a linear combination of the genotype data and a normalized weight function is constructed. To capture the genetic variations in the genotype function, the weight function is chosen to maximize the variance of *f*. This setting results in an optimization problem that can be transformed to a PCA or an eigenfunction problem. Therefore, the solution delivers not only the optimal weight functions but also principal component functions for the genotype data of the considered ROI. Because the optimization problem consists of integral functions and is difficult to solve in closed form, a solution is derived by discretizing the continuous eigenanalysis problem. Finally, principal component scores are constructed using the derived principal component functions and the genotype data. These then form the basis of the final FPCA test statistic, which considers the mean squared distance of averages of these principal components scores in cases and controls.

### Combined multivariate and collapsing method

The CMC method combines collapsing with a multivariate test [6]. The group of variants is divided into subgroups on the basis of predefined criteria, such as allele frequencies. The variants within each subgroup are collapsed, and a multivariate test, such as Hotelling's T^2 ^test or Fisher's product method, is applied for the analysis of all groups of variants together. In this analysis, Fisher's product method was used.

### Material

We applied both methods to case-control data provided for GAW18. Genotypes were provided for odd-numbered autosomes, but we dropped chromosome 5 data because of quality issues. We considered the simulated dichotomous phenotype of hypertension (HTN) in the sample of unrelated individuals and defined those individuals as cases who were defined as affected at least once at any time point of investigation. Controls were defined as the complement set of the cases. In the original data set, there were 157 unrelated individuals. However, only data from 142 of these individuals were used by the GAW18 organizers to create the 200-replicate data set. Because of the definition of case and control status with longitudinal data, the total numbers of cases and controls differed for each replicate, but in median, there was a ratio of cases to controls of about 0.84 over all replicates. Analyses were restricted to minor allele counts, so dosage files were used.

Gene information data was obtained by merging single-nucleotide polymorphism (SNP) data with the ENSEMBL database [9]. In total, there were 8,348,674 SNPs, of which 4,017,987 could be matched to ENSEMBL data. Furthermore, data merging resulted in 15,578 genes, of which 14,525 were included in the analysis because they had at least two variants; those containing only monomorphic variants were dropped. On the basis of these assumptions, we dropped 4 of the original 54 associated genes of the simulated data.

For our analyses, we used a modified version of the FPCA function provided by Luo *et al *[7] in R. All further analyses were done in R version 2.15.0 [10]. For CMC, variants were collapsed with a minor allele frequency (MAF) of 0.05 or less, and variants with a larger MAF were investigated separately for each gene. The global significance level was set to 0.05.

## Results

**Figure 1:**
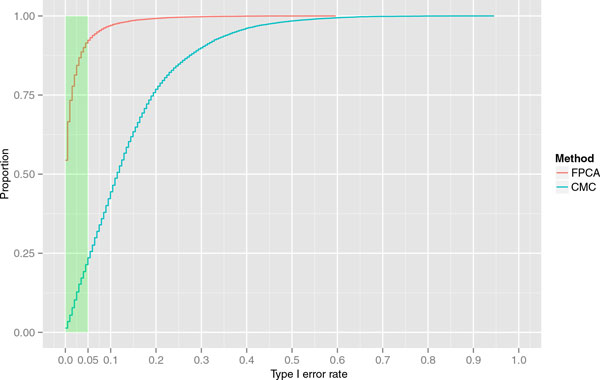
**Type I error rate with respect to proportion of unassociated genes of combined multivariate and collapsing (CMC) method and the functional principal component analysis (FPCA)**. Cumulative proportion of unassociated genes exceeding a given type I error using the CMC method and FPCA.

Figure 1 shows the cumulative proportion of unassociated genes exceeding a given type I error using CMC and FPCA. It can be seen that for FPCA, about 92% of the genes are below a false-positive rate of 0.05, but this is true for only about 25% of the genes using CMC. The maximum type I error of a single gene is about 0.88 in CMC and 0.55 in FPCA.

**Figure 2:**
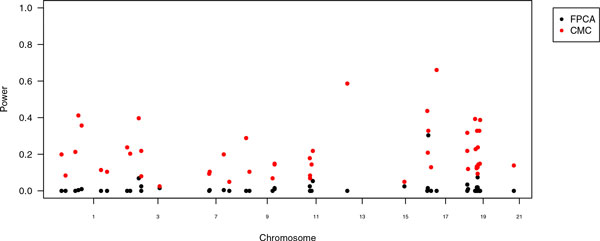
**Power of the combined multivariate and collapsing (CMC) method and the functional principal component analysis (FPCA)**. Power of the CMC method and FPCA in a Manhattan-like plot for all odd-numbered autosomes but chromosome 5.

Figure 2 shows the power for both methods. Whereas CMC had its highest power of 0.66 for gene *KRT23*, FPCA had its highest power in gene *SAT2 *with 0.30. The second highest power of FPCA is in gene *DBP *with 0.075. Table 1 shows the genes that had a power of 0.05 or greater in both methods. The most frequently identified gene by both methods simultaneously was *SAT2*. The power of FPCA to detect this gene was 0.3; for CMC, it was 0.33.

**Table 1:** Power of top genes for both methods.

Gene	Begin	End	No. of variants	Chromosome	FPCA	CMC
***MAP4***	47892181	47951731	4694	3	0.070	0.398
***GAB2***	77926342	78052926	3342	11	0.055	0.219
***SAT2***	7529551	7531173	23	17	0.303	0.328
***DBP***	49133819	49140639	33	19	0.075	0.095

Power of top four genes, with gene information, where power of combined multivariate and collapsing (CMC) method and of the functional principal component analysis (FPCA) is 0.05 or greater.

## Discussion

In this study, we compared two different collapsing approaches using the GAW18 data. We considered the simulated dichotomous phenotype HTN among the unrelated individuals without any restriction to MAF or covariates. The proportion of unassociated genes exceeding a given type I error of 0.08 for FPCA was moderate, but CMC's corresponding proportion of 0.75 was highly unacceptable. The power of both methods was too low for identifying most of the truly associated genes. Because CMC had a high false-positive rate, it cannot be used reliably for judging power. Its greatest power of 0.66 was observed for the gene *KRT23*. Table 1 illustrates that both methods fail in identifying associated genes in the simulated data set. Possibly, a strict filtering of variants by small MAFs would lead to better performance. Furthermore, the exclusion of best-guess genotypes or the inclusion of knowledge about functionality of the variants could be helpful.

## Competing interests

The authors declare that they have no competing interests.

## Authors' contributions

AZ designed the overall study, CD and AS conducted statistical analyses and CD and IRK drafted the manuscript. All authors read and approved the final manuscript.
